# Initial Evaluations of the Microtubule-Based PET Radiotracer, [^11^C]MPC-6827 in a Rodent Model of Cocaine Abuse

**DOI:** 10.3389/fmed.2022.817274

**Published:** 2022-02-28

**Authors:** Naresh Damuka, Thomas J. Martin, Avinash H. Bansode, Ivan Krizan, Conner W. Martin, Mack Miller, Christopher T. Whitlow, Michael A. Nader, Kiran Kumar Solingapuram Sai

**Affiliations:** ^1^Department of Radiology, Wake Forest School of Medicine, Winston-Salem, NC, United States; ^2^Department of Anesthesiology, Wake Forest School of Medicine, Winston-Salem, NC, United States; ^3^Department of Physiology and Pharmacology, Wake Forest School of Medicine, Winston-Salem, NC, United States

**Keywords:** PET imaging, radiochemistry, MPC-6827, cocaine addiciton, microtubules (MTs)

## Abstract

**Purpose:**

Microtubules (MTs) are structural units made of α and β tubulin subunits in the cytoskeleton responsible for axonal transport, information processing, and signaling mechanisms—critical for healthy brain function. Chronic cocaine exposure affects the function, organization, and stability of MTs in the brain, thereby impairing overall neurochemical and cognitive processes. At present, we have no reliable, non-invasive methods to image MTs for cocaine use disorder (CUD). Recently we reported the effect of cocaine in patient-derived neuroblastoma SH-SY5Y cells. Here we report preliminary results of a potential imaging biomarker of CUD using the brain penetrant MT-based radiotracer, [^11^C]MPC-6827, in an established rodent model of cocaine self-administration (SA).

**Methods:**

Cell uptake studies were performed with [^11^C]MPC-6827 in SH-SY5Y cells, treated with or without cocaine (*n* = 6/group) at 30 and 60 min incubations. MicroPET/CT brain scans were performed in rats at baseline and 35 days after cocaine self-administration and compared with saline-treated rats as controls (*n* = 4/sex). Whole-body post-PET biodistribution, plasma metabolite assay, and brain autoradiography were performed in the same rats from imaging.

**Results:**

Cocaine-treated SH-SY5Y cells demonstrated a ∼26(±4)% decrease in radioactive uptake compared to non-treated controls. Both microPET/CT imaging and biodistribution results showed lower (∼35 ± 3%) [^11^C]MPC-6827 brain uptake in rats that had a history of cocaine self-administration compared to the saline-treated controls. Plasma metabolite assays demonstrate the stability (≥95%) of the radiotracer in both groups. *In vitro* autoradiography also demonstrated lower radioactive uptake in cocaine rats compared to the control rats. [^11^C]MPC-6827’s *in vitro* SH-SY5Y neuronal cell uptake, *in vivo* positron emission tomography (PET) imaging, *ex vivo* biodistribution, and *in vitro* autoradiography results corroborated well with each other, demonstrating decreased radioactive brain uptake in cocaine self-administered rats versus controls. There were no significant differences either in cocaine intake or in [^11^C]MPC-6827 uptake between the male and female rats.

**Conclusions:**

This project is the first to validate *in vivo* imaging of the MT-associations with CUD in a rodent model. Our initial observations suggest that [^11^C]MPC-6827 uptake decreases in cocaine self-administered rats and that it may selectively bind to destabilized tubulin units in the brain. Further longitudinal studies correlating cocaine intake with [^11^C]MPC-6827 PET brain measures could potentially establish the MT scaffold as an imaging biomarker for CUD, providing researchers and clinicians with a sensitive tool to better understand the biological underpinnings of CUD and tailor new treatments.

## Introduction

Imaging is an important tool for studying neuropathologic, morphologic, and functional changes associated with cocaine use disorder (CUD). While receptor- and neurotransmitter-based positron emission tomography (PET) imaging biomarkers are being investigated to understand neurochemical, biological and treatment strategies, sensible and quantifiable non-traditional imaging biomarkers are needed and cytoskeleton-based biomarkers could be a promising candidate. Microtubules (MTs) are hetero-dimer units of the cytoskeleton formed from α and β-Tubulin monomers (1). Their structural integrity and polymerization process between bound and free α/β tubulin units is critical for key biophysical functions including cellular signaling and axoplasmic transport, which are also essential for the reward circuit system that drives addiction in several substance use disorders including cocaine (2–5). Repeated exposure to cocaine induces structural plasticity in the brain reward circuits, with significant consequences, including behavioral changes and cognitive deficits (6, 7). It alters dendritic spine morphology (8) and density through varied MT and actin rearrangements in the nucleus accumbens and prefrontal cortex (8–11). Additionally, MT impairments have also been linked to some behavioral and cognitive deficits in CUD subjects. Despite the importance of MT changes in CUD, their underlying *in vivo* mechanisms remain largely unknown.

Recently, we reported the automated radiochemical synthesis of [^11^C]MPC-6827 as the first brain-penetrating MT PET ligand and demonstrated its *in vivo* PET imaging utility in cocaine-naïve rodents and non-human primates (12, 13). Cell uptake results in a patient-derived neuroblastoma SH-SY5Y cells with cocaine treatment showed that [^11^C]MPC-6827 binds selectively to destabilized tubulin units (14). Therefore, direct *in vivo* imaging of MT levels could reveal real-time cytoskeletal changes during CUD progression. In this project, we report our preliminary results of [^11^C]MPC-6827 in a rodent model of CUD using *in vivo* imaging and *ex vivo* biodistribution and autoradiography studies. A well-established rat model of cocaine self-administration (SA) was selected to track MT integrity and correlate with cocaine intake (15, 16).

## Materials and Methods

### Chemicals

MPC-6827 hydrochloride (Cat. # 5231) was purchased from Tocris (Minneapolis, MN, United States) and all the anhydrous reagents and solvents were purchased from Sigma Aldrich (St. Louis, MO, United States). The MT/tubulin assay kit (Cat. # BK038) was obtained from Cytoskeleton, Inc. (Denver, CO, United States) Desmethyl MPC6827 (precursor for [^11^C]MPC-6827 radiochemistry) was purchased from ABX biochemical compounds (Radeberg, Germany). Cocaine HCl was provided by the Drug Supply Program of the National Institute on Drug Abuse (Rockville, MD, United States).

### Animals

All female and male rats were purchased from Envigo (Raleigh, NC, United States) and were 7 weeks of age on arrival. Animals were given ad lib access to standard rat chow (ProLab RMH 3000, LabDiet, Brentwood, MD, United States) and water except during experimental conditions. All animal experiments were conducted following the Institutional Animal Care and Use Committee-approved protocols in compliance with the Guidelines for the Care and Use of Research Animals established by the Wake Forest University Health Sciences (WFUHS) Animal Studies Committee.

### [^11^C]MPC-6827 Radiochemistry

Radiosynthesis of [^11^C]MPC-6827 was validated and optimized in GE-FX2MeI/FX2M radiochemistry module. Briefly, [^11^C]MeI was bubbled into the corresponding desmethyl MPC-6827 (0.6 ± 0.1 mg) precursor in 5N NaOH solution (10 μL) in DMF (0.4 mL) at 80°C for 5 min. The reaction mixture was injected onto a semi-preparative HPLC separation with a reverse-phase C18 column (Phenomenex, 250 × 10 mm, 10 μ), UV at 254 nm, and a flow rate of 5 mL/min. Desired radioactive product was collected in a 50 mL preloaded water vial and passed through C18 sepPak cartridge (WAT036800, Waters, Milford, MA, United States). The final product [^11^C]MPC-6827 was eluted with 10% ethanol in saline solution into a sterile vial *via* a 0.2 μm filter for further *in vitro* and *in vivo* studies.

### Cell Binding Assays

We recently demonstrated that cocaine treatment in patient-derived SH-SY5Y neuroblastoma cells changes the MT integrity (14). Cocaine treated cells showed ∼24% fewer free/unbound tubulin units than the untreated cells. [^11^C]MPC-6827-based cell binding assays were performed in SH-SY5Y cells *in vitro* following our published protocols (17). Briefly, cells were treated with 1 mM cocaine for 3 days, and [^11^C]MPC-6827 (0.074 MBq/well) was added and incubated for 30 and 60 min (*n* = 6/time point). To demonstrate tracer specificity, a subgroup of cells (*n* = 6) was pre-treated with non-radioactive MPC-6827 (1.0 μM), and 60 min later, radiotracer was added and incubated for 30 min. All cells were washed with PBS and lysed with 1N NaOH. Finally, the lysate from each well was γ-Counted, and counts-per-minute (cpm) values were normalized to the amount of radioactivity added to each well. Cpm values were then matched with the protein concentration per well, and the data expressed as% injected dose/mg (ID/mg) of protein in each well.

### Cocaine Self-Administration

Fisher F-344 rats (*n* = 4/sex) with chronic jugular catheters were trained to intravenously SA cocaine for 5 weeks as previously described (15, 18). Cocaine availability was signaled by illumination of stimulus light above the active lever. Each lever press delivered an infusion of 1.5 mg/kg of cocaine in 0.2 mL of heparinized saline (0.9% w/v NaCl, pH 7.4 with 1.7 U/mL heparin sodium), signaled by operation of a house light and tone, and was followed by a 20 s time-out period during which lever presses had no programmed consequences. The operant chambers were housed in sound and light-attenuating cubicles, and 2 h SA sessions were conducted 5 days per week during the dark phase of the light: dark cycle (dark 05:00–17:00). The control rats (*n* = 4/sex) received hourly infusions of 0.2 mL heparinized saline in their home cages. Cocaine SA rats received hourly infusions of heparinized saline when not in SA sessions.

### PET Imaging in Cocaine Self-Administration Rats

[^11^C]MPC-6827 dynamic 60 min brain PET/CT imaging was conducted in cocaine SA and saline-treated control rats (*n* = 4/sex/group). All 16 rats received baseline scans with [^11^C]MPC-6827 (∼18.5 ± 0.01 MBq) *via* intravenous tail injection. Eight rats (*n* = 4/sex) underwent cocaine self-administration, and the remaining 8 (*n* = 4/sex) received *iv* saline for 5 weeks and were re-scanned with [^11^C]MPC-6827 (24 h after the last cocaine SA session) with the same parameters. The PET images were constructed by a TriFoil-based algorithm into dicom images and the images (both PET and CT) were processed to the PMOD software system using the TriFoil built-in software program. The list mode of the emission scans were reframed into a dynamic sequence of 1 × 3 s, 6 × 2s, 9 × 5s, 6 × 10s, 4 × 30 s, 2 × 60 s, 2 × 2 min, and 10 × 5 min frames. Images were reconstructed using PMOD software (PMOD technologies). The 3D regions of interest (ROIs) were manually drawn for the whole brain as contours in high-resolution PET images. Time Activity Curves (TACs) were obtained using the PMOD-generated numbers and were expressed as standardized uptake values (SUVs) from the fused microPET/CT images. The parameter SUV is defined as tissue radioactivity concentration [MBq/g] × body weight (g)/injected dose [MBq].

### Post-PET Biodistribution Studies

Post-PET radiotracer tissue biodistribution studies were performed with [^11^C]MPC-6827 in the same rats after PET/CT acquisition. All the rats were euthanized and radioactive uptake in the brain, blood, heart, lungs, liver, kidney, spleen, pancreas, and muscle was calculated as percentages of injected dose per gram of tissue (%ID/g tissue) and with a standard dilution of the injectate. Radiotracer uptakes in the tissues were measured using a γ-Counter (12) and expressed in %ID/g tissue.

### Metabolic Profile in Plasma

To determine the metabolic stability of [^11^C]MPC-6827 *ex vivo*, plasma HPLC-based metabolite assays were performed in the same cocaine SA and control rats (24 h-post-treatment) following published methods (19, 20). Briefly, whole blood (∼0.5 mL) was quickly collected (∼60 min-post PET scans) during the biodistribution studies, γ-Counted and centrifuged to separate red blood cells from plasma. A portion of clear plasma was treated with ice-cold acetonitrile and re-centrifuged and the supernatant fraction was γ-Counted, and injected into a WATERS analytical HPLC system equipped with an Agilent C18 column (250 × 4.6 mm, 5 μ), UV_*max*_ @ 254 nm. The HPLC fractions were collected at 1 min intervals for 15 min and each fraction was γ counted and decay-corrected.

### Autoradiography Studies

Autoradiography studies *in vitro* were performed on the frozen brain tissues 24 h post-biodistribution studies to primarily correlate *in vivo* PET imaging measures following the published protocols. Briefly, the coronal sections from the frozen brain tissues (48 h-post last cocaine SA session) were mounted on glass slides (Super Frost Plus slides), and air-dried for 30 min, and incubated in PBS (pH 7.4) for 10 min. [^11^C]MPC-6827 in PBS (∼5 MBq) was added to each slide and incubated for 30 min. The slides were then washed with PBS (3X) and water (1X) at 4°C, and quickly air-dried. Slides containing the radioactive brain tissues were exposed to a radioluminographic imaging plates and scanned with GE Amersham Typhoon scanner. Autoradiographs were analyzed using ImageQuant TL 8.2 and uptake was calculated as phosphor-stimulated luminescence (PSL/mm^2^).

### Statistical Analysis

All the data including, cell uptake, microPET imaging, biodistribution, and autoradiography studies were reported as the average value ± standard deviation. Statistical analysis was performed using a two-tailed Student’s *t*-test with *p < 0.05, being considered significant.

## Results

### Radiochemistry

[^11^C]MPC-6827 was produced (12) with a radiochemical yield of ∼38 ± 5%, radiochemical purity of >95%, and molar activity of ∼144,300 ± 100 MBq/μmol, decay corrected to the end of synthesis (*n* > 55 runs) (12, 13, 21–23).

### Cell Uptake Studies

**FIGURE 1:**
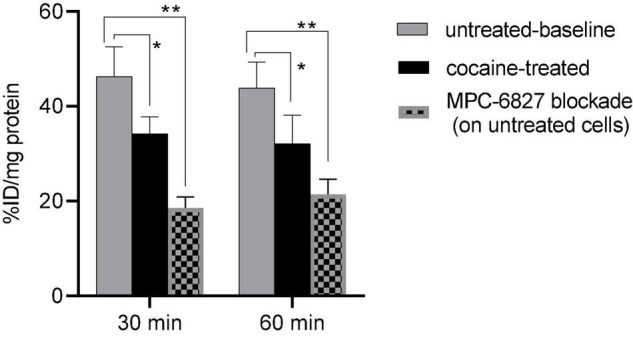
[^11^C]MPC-6827 uptake *in vitro* at 30 and 60 min incubation times with and without cocaine, and MPC-6827 blockade treatments (*n* = 6/group) in SH-SY5Y cells; *p ≤ 0.05, **p = 0.007.

Cocaine-treated cells demonstrated a ∼26(±4)% decrease in radioactive uptake compared to non-treated controls over the 30–60 min incubation times (Figure 1). In self-blocking assays, uptake was ∼60(±4)% lower after we added non-radioactive MPC-6827, demonstrating high specificity. These blocking studies might show some degree of non-specific binding, which is commonly associated with both routine and novel radiopharmaceutical evaluations in PET imaging (24). This is likely due to *in vitro* artifacts or not completely saturating target site. These assays are used to demonstrate a proof-of-principle specificity, which often do not show complete blocking. MPC-6827 primarily targets the β tubulin sites at pharmacological doses (14, 25, 26). The lowered radioactive uptake in cocaine-treated SH-SY5Y cells indicates that [^11^C]MPC-6827 may be tracking the destabilized free β tubulin units, as cocaine treatment decreased free tubulin content in the same cells.

### Cocaine Self-Administration

Daily cocaine intake (mean ± SD) over 5 weeks of SA in male rats (*n* = 4) was 84.2 ± 2.91 mg/kg/d and in female rats (*n* = 4) was 88.3 ± 7.1 mg/kg/d (*p* = 0.4). The control rats (*n* = 4/sex) received hourly saline infusions.

### MicroPET Imaging Studies

**FIGURE 2:**
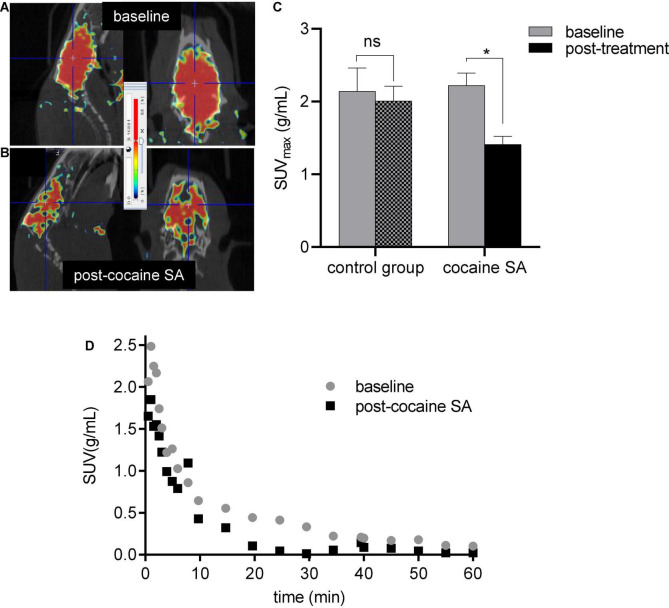
Representative microPET/CT image of a cocaine SA rat brain at **(A)** baseline and **(B)** post-cocaine treatment with [^11^C]MPC-6827 and their whole-brain **(C)** SUV_*max*_ and **(D).** TACs; ns, non-significant, *p = 0.043.

Radiotracer demonstrated high brain uptake with good retention in both groups. TACs showed radioactivity peaked at ∼4–5 min followed by a gradual washout by 60 min in both cocaine self-administered and control rat groups; however, the cocaine SA group demonstrated 1.3-fold lower brain uptake over the control group. Whole-brain SUV_*max*_ in rats self-administering cocaine (2.16 ± 0.1) showed ∼33(±2)% less uptake than at baseline (1.41 ± 0.01), while control rats showed no significant difference (Figure 2) in their radioactive brain uptakes (2.14 ± 0.08 vs. 1.99 ± 0.1). No significant differences were seen between the male and female rats in both the control and cocaine SA groups.

### Biodistribution Results

**FIGURE 3:**
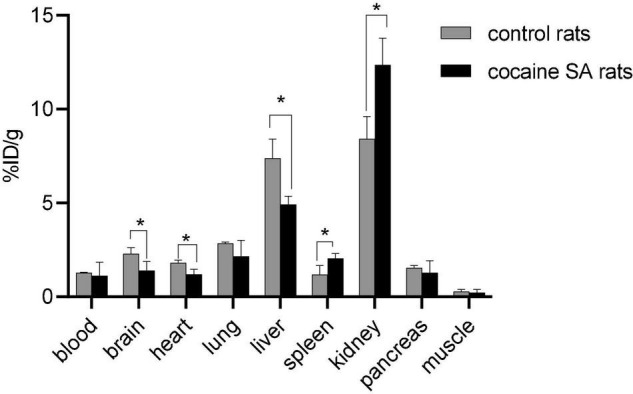
Post-PET biodistribution profile of [^11^C]MPC-6827 in control and cocaine SA rats (*n* = 4/sex); *p ≤ 0.05, **p = 0.0038.

Rats that self-administered cocaine (%ID/g = 2.28 ± 0.15) had ∼49% lower [^11^C]MPC-6827 brain uptake than control rat uptake (%ID/g = 1.39 ± 0.22). The favorable kinetics of [^11^C]MPC-6827, including washout of radioactivity from peripheral organs, renal (%ID/g = 8.37 ± 1.78) and/or hepatic (%ID/g = 7.14 ± 1.63) clearance, and non-significant muscle background (%ID/g = 0.19 ± 0.07) supports its high translational utility (Figure 3). Again, no significant differences in radiotracer distribution were seen between male and female rats.

### Metabolic Profile in Plasma

**FIGURE 4:**
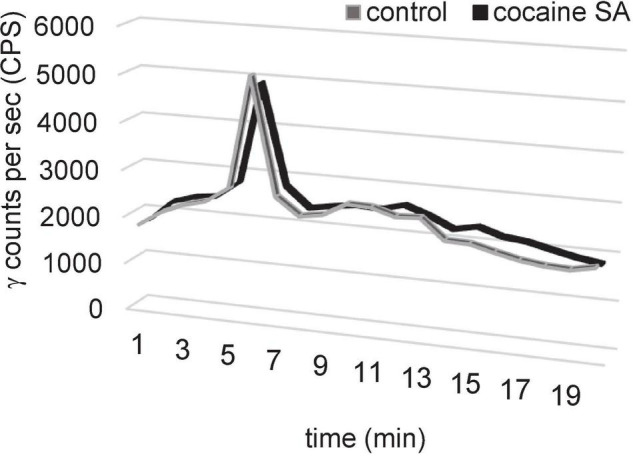
Representative HPLC plasma metabolite results of [^11^C]MPC-6827 in a control and cocaine SA rat demonstrating a major peak (>95%) at a retention time of 5.3 min of parent ([^11^C]MPC-6827) peak after ∼65 min of radiotracer injection.

The plasma sample recovery yield, HPLC extraction efficiency, and fraction recovery were ≥95% (Figure 4). The HPLC radiochromatogram in plasma samples from cocaine SA and control rats indicated that the parent radiotracer, [^11^C]MPC-6827, had a retention time of 5–6 min and was the major constituent (>95%).

### Autoradiography Studies

**FIGURE 5:**
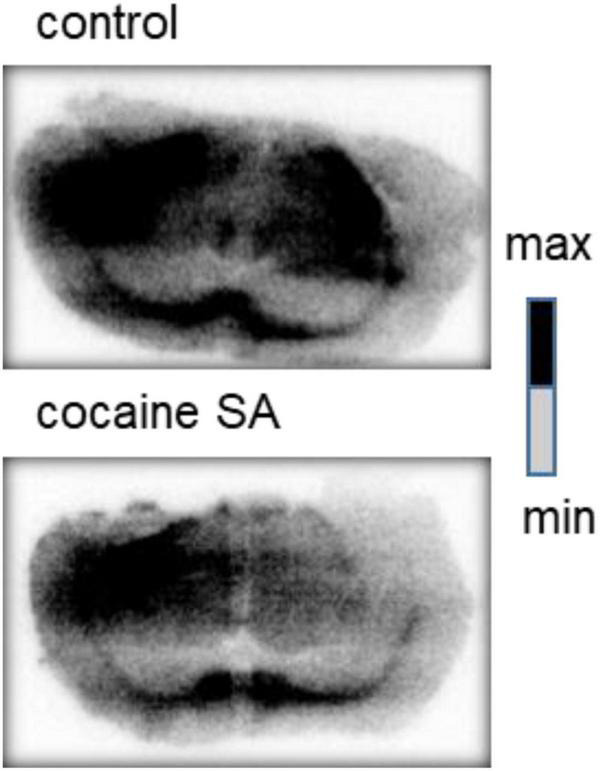
Representative *in vitro* autoradiography images of a control and cocaine SA rat coronal brain sections with [^11^C]MPC-6827. We used George paxnos and charles watson in stereotaxic coordinates for the brain coronal sections, with the visible brain structures in the images are the corpus callosum and striatum.

*In vitro* autoradiography images also demonstrated lower radioactive of [^11^C]MPC-6827 in cocaine SA rat brain tissues over the saline-treated controls (Figure 5). Importantly, lower radioactive uptake was seen in the prefrontal cortex and striatum regions of the cocaine SA rat brains.

## Discussion

Microtubules are the structural units of neurons responsible for axonal transport, information processing, and signaling mechanisms. Polymerization of α and β tubulin subunits forms the MT cytoskeleton and chronic cocaine exposure may alter the integrity of both bound and unbound (free) tubulin forms to induce morphological and functional changes in cytoskeleton and neurons. Therefore, direct *in vivo* imaging of MT levels could reveal real-time cytoskeletal changes during CUD progression. MPC-6827 binds to MTs with high affinity (IC_50_ = 1.5 nM), acting primarily on colchicine-binding β tubulin sites, and has proven safe for use in human subjects in clinical trials for glioblastoma. Most important, MPC-6827 has ideal *in vivo* pharmacokinetics (25, 27–30). Although, clinical outcomes suggest that MPC-6827 may have limited success in cancer therapy (31, 32), its corresponding radiolabeled analog has potential as a CNS PET imaging agent based on blood-brain-barrier (BBB) penetration, on-target specific binding, and a favorable safety profile in humans. Our laboratory reported the first brain penetrant MT-based PET imaging agent, [^11^C]MPC-6827, and evaluated it’s *in vivo* efficacy in cocaine-naïve rats (12) and non-human primates (13).

Cells maintain a balance between stabilized polymerized MTs and destabilized α/β free tubulin units in the cytoplasm (33). We recently reported [^11^C]MPC-6827’s *in vitro* selectivity toward destabilized tubulin units using various MT agents including stabilizing and destabilizing agents in patient-derived SH-SY5Y neuroblastoma cells (14). Using MT-based assay (Cytoskeleton Inc, Denver, CO) cocaine-treated cells showed ∼21% less destabilized MT content than untreated cells, indicating that cocaine exposure compromises cellular MT stability, (34–36) treated with cocaine and untreated cells (14). After demonstrating changes in MT integrity *in vitro* with cocaine, our next step was to determine whether [^11^C]MPC-6827 could detect the same MT alterations in the same cells. Cell-binding assays were performed in SH-SY5Y cells *in vitro* with [^11^C]MPC-6827 following our published protocols (17, 37). Cocaine-treated cells showed ∼26% lower [^11^C]MPC-6827 uptake compared to non-treated controls over the 30–60 min incubation times. The lowered radioactive uptake in cocaine-treated SH-SY5Y cells indicates that [^11^C]MPC-6827 may be tracking destabilized/free β tubulin units, as cocaine treatment decreased destabilized/free tubulin content in the same cells and MPC-6827 binds to colchicine sites of β tubulin units.

After successfully evaluating the *in vitro* potential of the radiotracer, the present study extended this work by examining [^11^C]MPC-6827’s *in vivo* imaging efficiency in a well-established rodent model of CUD, intravenous cocaine self-administration (SA). This model (38–40) allowed for the assessment of MT stability during stable cocaine SA (15, 16) and to compare the changes against age-matched controls (saline-treated rats). We hypothesize that PET imaging with [^11^C]MPC-6827 in a rodent model of CUD will provide critical information on the MT changes in the brain during cocaine use. All the rats received baseline dynamic 0–60 min brain PET/CT acquisition with [^11^C]MPC-6827 (∼18.5 MBq tail intravenous injection). Eight rats (*n* = 4/sex) underwent cocaine SA, and the remaining 8 (*n* = 4/sex) received *iv* saline for 5 weeks and re-scanned with [^11^C]MPC-6827. SUV_*max*_ demonstrated a ∼33(±2)% decrease in radioactive uptake in rats that self-administered cocaine vs. control animals. To limit biological variability, we included equal number of male and female rats. While previous literature (41, 42) showed some differences between male and female cocaine uptake in rodent models, there was no significant difference in [^11^C]MPC-6827 uptake between the male and female rats.

Standard biodistribution studies were primarily performed to determine if the *in vivo* PET imaging measures correlate with the *ex vivo* profile. Rats who self-administered cocaine (%ID/g = 1.49 ± 0.15) displayed ∼49% lower brain uptake compared to the controls (%ID/g = 1.49 ± 0.22). [^11^C]MPC-6827 demonstrated favorable radioactivity washout from peripheral organs, renal and/or hepatic clearance, and non-significant background uptake. There are some significant differences in radiotracer uptake in peripheral organs, which could be attributed to effect of cocaine on other organs, with some expression of MTs. While comprehensive multiple time-point biodistribution studies are needed to fully understand the pharmacokinetics of [^11^C]MPC-6827, our 60 min post-PET biodistribution results support its high translational utility. Importantly, *ex vivo* biodistribution results corroborate with the *in vivo* microPET, and *in vitro* cell uptake data. Cocaine SA decreased radioactive uptake, corresponding to the decreased availability of destabilized tubulins.

Preliminary metabolic stability of [^11^C]MPC-6827 was performed in cocaine self-administered and control rat plasma samples (*n* = 4/sex), 60 min post-PET imaging. The parent radiotracer [^11^C]MPC-6827 was the major (96 ± 2%) constituent in samples from both groups. Minor peaks (3.5 ± 1%) were seen with retention times earlier than the parent radiotracer, indicating a potential lipophilic metabolite. High-resolution mass spectral analysis will be required to further characterize the complete metabolite profile. These initial data suggest that radiotracer was metabolically stable in plasma *ex vivo*, ∼60 min-post radiotracer injection and the profile was similar in both cocaine SA and control rats.

Autoradiography (ARG) is commonly performed to validate *in vivo* imaging results and identify the target and non-target regions of high radioactive uptake. [^11^C]MPC-6827 *in vitro* ARG results showed lower radioactive uptake in the pre-frontal cortex and striatum (dorsal and ventral) of cocaine SA rats compared to control rats, demonstrating regional specificity of the radioligand.

Cocaine-mediated cognitive impairments are primarily caused by dynamic cytoskeletal rearrangements involved in mediating structural and behavioral plasticity (43, 44). At a molecular level, cocaine-induced synaptic activity triggers diverse signaling pathways, which regulate cytoskeleton-associated protein reorganization pathways, which ultimately led to changes in dendritic structure (33). At a structural level, chronic cocaine exposure is also associated with dendritic spine architecture for which actin polymerization is necessary—for which microtubule (MT) dynamics is essential (45). Additionally, cocaine SA paradigm changes the MT dynamics by binding at few microtubule-associated proteins (MAPs) at their plus end (6). In our previous work, we demonstrated that stabilized MTs decrease and destabilized increase [^11^C]MPC-6827 uptake (14). Collectively, cocaine administration could change MT dynamics through alterations of MAP binding sites which could result in less destabilized MTs resulting in less radioactive uptake. Our next steps would be performing a comprehensive, multi-timepoint blood-based metabolic studies of the radiotracer in both control and cocaine self-administrated rats.

## Conclusion

Our preliminary findings demonstrate that [^11^C]MPC-6827 demonstrated lower uptake in cocaine-treated SHSY-5Y cells over untreated cells *in vitro* and reduced brain uptake in rats that were self-administering cocaine relative to control rats *in vivo* and *ex vivo*. Additionally, plasma metabolite results showed high stability of the radiotracer ∼60 min-post injection in both cocaine and control groups. These preliminary studies are the first steps to demonstrate changes in MTs *in vitro, in vivo*, and *ex vivo* in a well-established rodent model of cocaine SA using [^11^C]MPC-6827. Future studies on the radiotracer’s complete metabolite profile, multiple timepoint biodistribution studies, and correlation of PET measures with different cocaine intakes will validate the utility of MTs as a neuroimaging biomarker for CUD.

## Data Availability Statement

The original contributions presented in the study are included in the article/supplementary material, further inquiries can be directed to the corresponding author.

## Ethics Statement

The animal study was reviewed and approved by the Wake Forest University Health Sciences (WFUHS) Animal Studies Committee.

## Author Contributions

KKSS designed and developed the entire research. TM, MN, and CW helped in designing the rodent experiments and image analyses. ND, AB, MM, IK, and CM performed radiochemistry, cell uptake, imaging, plasma assay, and autoradiography studies. ND, MN, TM, and KKSS analyzed the data. ND, MM, IK, and KKSS wrote the manuscript. All authors reviewed the manuscript. All authors contributed to the article and approved the submitted version.

## Conflict of Interest

The authors declare that the research was conducted in the absence of any commercial or financial relationships that could be construed as a potential conflict of interest.

## Publisher’s Note

All claims expressed in this article are solely those of the authors and do not necessarily represent those of their affiliated organizations, or those of the publisher, the editors and the reviewers. Any product that may be evaluated in this article, or claim that may be made by its manufacturer, is not guaranteed or endorsed by the publisher.
